# Confident judgments of (mis)information veracity are more, rather than less, accurate

**DOI:** 10.1093/pnasnexus/pgag186

**Published:** 2026-06-30

**Authors:** Akshina Banerjee, Matthew D Rocklage, Mohsen Mosleh, David Rand

**Affiliations:** Department of Marketing, University of Michigan, Ann Arbor, MI 48109, USA; Department of Marketing, Northeastern University, Boston, MA 02115, USA; Oxford Internet Institute, University of Oxford, Oxford OX2 6GG, United Kingdom; Department of Marketing, Cornell University, Ithaca, NY 14853, USA

## Abstract

Does confidence help or hinder the recognition of misinformation? Prior work has reached opposing conclusions, in part because it has not separated confidence in a specific judgment from confidence in one's abilities in general. Here, we separate these constructs and test them side-by-side. In a large, preregistered study on Lucid (*n* = 503) where participants judged the accuracy of news headlines, confidence in specific judgments predicted greater accuracy. In contrast, general confidence predicted greater “inaccuracy.” In a preregistered prolific replication (*n* = 498), the confidence-in-judgment effect replicated, whereas the confidence-in-general effect was less consistent. Together, these results indicate that when people say they are confident in a specific judgment, they tend to be right—but people who are generally confident in themselves are no better at spotting misinformation and may even be worse.

## Introduction

In recent years, the issue of misinformation has garnered significant attention due to concerns about its influence across various sectors, from public health to politics. One main stream of research in this area has focused on understanding factors that are associated with the (in)ability to tell truth from falsehood. For example, people who engage in more deliberation/analytic thinking are better at telling true from false (1), and, similarly, people with higher levels of media and digital literacy are better at discerning between true and false information (2, 3).

Here, we focus on another factor that has been implicated in the ability to discern truth: overconfidence. Prior work has found that people who are more confident in their abilities to tell true from false are in fact worse at doing so (4) and are more likely to share low-quality news (5). Similarly, people who are more confident in their own cognitive abilities are more likely to believe conspiracy theories and to believe that others also believe such theories (6). The natural conclusion has been that confidence in one's general abilities is an important contributor to misbelief—yet that conclusion rests largely on measures of general confidence, rather than confidence in specific judgments.

One's level of general confidence in one's abilities, however, is not the same as the confidence people have in a “specific” judgment. One may be generally confident in their abilities but still experience variation in confidence when evaluating specific pieces of information—and confidence in specific judgments might play a very different role in discerning between true and false news than general confidence. In particular, variation in confidence across judgments may provide individuals with a relatively accurate signal of the true validity of information. Consistent with this proposal, a recent study found that participants’ confidence in a specific judgment often tracks their actual accuracy in detecting misinformation—and thus that more confident judgments had higher accuracy discernment (7, 8). It is difficult, however, to conclude that general and specific confidence have different associations with truth discernment based on the existing evidence—prior studies varied not only in which form of confidence they measured but also many other aspects of the experimental design, including the headlines used, the subject pool from which participants were drawn, and the time when the studies were run. Thus, it is possible that these differences drove the difference in association between confidence and discernment, rather than fundamental differences between general and specific confidence. Furthermore, to our knowledge, only one study each has examined how discernment relates to general (5) or specific (7) confidence. Thus, the replicability of the prior findings remains untested, and another possible explanation for divergent findings is simply that one of the two prior results was a false positive. Therefore, in the current work, we jointly evaluate how confidence in general and confidence in specific judgments relate to the ability to discern truth from falsehood within the same study. Beyond testing both constructs in one short study, we also assess their robustness in our setup: we evaluate which construct—confidence in judgment or confidence in general—exhibits the more stable association with veracity judgments.

## Results

**Figure 1:**
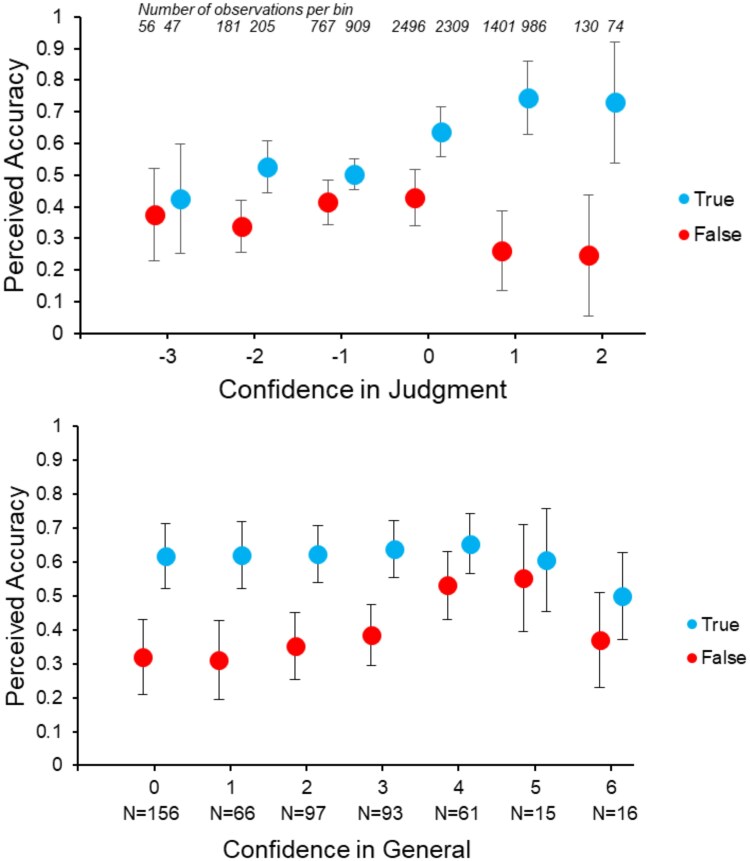
Confidence in judgment and confidence in general show opposite relationships with accuracy discernment. Shown is the average perceived accuracy rating of true and false headlines as a function of (top) the participant's confidence in that specific accuracy judgment, relative to the user's average confidence across headlines (i.e. de-meaned—for clarity, we omit −4 and +3 bins for false because those bins had only two participants each); and (bottom) the participant's confidence in their general abilities. Error bars indicate 95% CIs clustered on subject and headline.

Our analyses reveal different effects of confidence in general versus confidence in judgment in predicting the veracity of news headlines. Confidence in judgment was elicited by asking participants how confident they were in their veracity judgment, while confidence in general was elicited using the “Generalized Overconfidence Task” (4), a politically neutral perceptual task designed to disconnect perceived performance from actual performance, allowing us to capture domain-general self-assessment tendencies unrelated to political beliefs or news discernment. We estimated a mixed-effects model predicting binary headline truth judgments from standardized headline-level confidence (confidence-in-judgment) and standardized participants’ standardized general confidence (confidence-in-general), binary headline veracity (false vs. true), and partisanship (centered), including all two- and three-way interactions among these predictors, with participant and headline as random intercepts and slopes. We found that higher confidence in general was associated with greater susceptibility to false news—people who had higher levels of general confidence were worse at discerning true from false headlines (interaction between confidence in general and false dummy, *γ* = 0.066, *t* = 3.51, *P* = 0.002). However, we also observe that confidence in judgment—that is, confidence about the truth judgments for each headline—showed the opposite pattern. That is, when participants had higher levels of confidence in their judgment about headline-level veracity, they were “better” at discerning true from false headlines (interaction between confidence in judgment and false dummy, *γ* = −0.096, *t* = −5.77, *P* < 0.001; see Fig. 1).

To understand what is driving these interactions, we examined the simple effects of each type of confidence separately for true and false headlines. The most consistent pattern involved confidence in judgment and true headlines: across analyses, confidence in judgment predicted greater perceived accuracy of true headlines (*γ* = 0.090, *t* = 8.57, *P* < 0.001), while confidence in general was not significantly related to perceived accuracy of true headlines (*γ* = −0.013, *t* = −0.99, *P* = 0.340). Effects for false headlines were more variable across samples and measures (see Supplementary material for full decomposition).

These findings were also replicated when using measures of general confidence in one's misinformation detection abilities from Lyons et al. (5) with the exception of the interaction between general confidence and the false dummy being marginally significant (*P* = 0.057), though still in the expected direction.

As another test of robustness, we conducted a replication using prolific (*n* = 498) with the same headline-judgment procedure. The key interaction between confidence-in-judgment and headline-level veracity replicated: higher confidence-in-judgment predicted increased belief in true headlines and decreased belief in false headlines (*γ* = −0.133, *t* = −8.70, *P* < 0.001). In contrast, the interaction between confidence-in-general and headline-level veracity was not statistically significant (*γ* = 0.016, *t* = 1.04, *P* = 0.309).

Taken together, these findings indicate that confidence-in-judgment is a robust predictor of misinformation discernment, primarily by predicting recognition of true headlines, and is distinct from confidence-in-general. The latter shows a less stable pattern—sometimes actually predicting “greater” susceptibility to false headlines.

## Discussion

These findings clarify seemingly conflicting results on confidence and misinformation detection by showing that confidence-in-judgment is the key construct linking confidence to accuracy. Across samples, higher confidence in specific truth judgments tracks better discernment—more belief in true headlines and less belief in false headlines—consistent with emerging work on metacognitive insight (7). In contrast, confidence-in-general is conceptually and empirically separable from confidence-in-judgment and is sometimes associated with worse discernment, echoing prior concerns about overconfidence (5). However, this association is less stable and may not emerge as often as the association between confidence-in-judgment and discernment.

The current study's aim was to demonstrate the conceptual distinction between general confidence and judgment-specific confidence when identifying misinformation. Future research can further examine the generalizability and implications of these findings. The platform we used to gather data (Lucid) has been shown to be more representative of the general population compared with other platforms (9). We also ran the study on prolific, which bolstered the basic pattern: confidence-in-judgment signals accuracy, whereas confidence-in-general does not, reinforcing that these constructs differ not only in sign but also in stability. Additionally, future experimental work could investigate whether interventions that target confidence in specific judgments (e.g. through calibration training) can improve actual accuracy or reduce misinformation sharing.

The implications of these results extend to both conceptual understanding and practice. Conceptually, we highlight the need for a more nuanced understanding of the role confidence plays in misinformation susceptibility. Beyond misinformation, our results emphasize the importance of differentiating different forms of confidence more generally. Practically, our findings highlight that interventions aimed at increasing truth discernment by reducing (over)confidence could also reduce confidence in judgment, potentially leading to backfires. Understanding the difference between these two types of confidence provides an approach to reducing belief in misinformation by encouraging individuals to rely on their confidence in each judgment individually, rather than relying on general confidence.

## Methods

We recruited participants (*n* = 503 after exclusions, preregistered) from Lucid. First, general confidence was measured in an unrelated “General Overconfidence Task” (4). Specifically, each participant was given an extremely difficult perceptual task where they were shown six fuzzy images and asked to guess what it was an image of by making a choice between two presented options. They were then asked to estimate how many of the images they got correct. This measure was designed to assess participants’ general confidence, independent of any specific skill set or domain knowledge. Given the difficulty of the task, participants’ perceptions of how well they did should be unrelated to how well they actually did (4)**)**—and indeed, we find actual performance was not significantly correlated with estimated performance (*r* = −0.02, *P* = 0.577). We calculated confidence in general as the estimated performance on the image task, providing a measure of confidence that is not tied to any particular skill set or domain knowledge.

Then, participants were shown 20 headlines in random order, out of which 10 were false and 10 were true. Moreover, 10 of the headlines in total were also partisan and the remaining were nonpartisan. For each headline they saw, participants were first asked to judge whether the headline was true (Yes/No format, later re-coded to be 1 for “yes” and 0 for “no” for the analyses). Then, they were asked to rate how confident they were in that judgment (1 = very unconfident to 7 = very confident).^a^

After they rated all 20 headlines, they were presented with the other general confidence questions from Lyons et al. (5), where they were asked to rate how they compared with other Americans at recognizing detecting news that is made up (0 = worse than 99% of people, 50 = equally bad/good as other, 100 = better than 99% of people) and then how they compared with other Americans in how well they performed in this study at recognizing news that is made up (0 = worse than 99% of people, 50 = equally bad/good as other, 100 = better than 99% of people). The *R*^2^ of the main model is reported. Finally, they answered a few demographic questions about themselves. See SI Appendix for exact wording for all items.

We also conducted a replication on prolific (*n* = 498 after exclusions, preregistered) using the same measures and the same analytic specification. We corrected the mistake from the previous study (see footnote) in this replication.

All studies were approved by the MIT Institutional Review Board (IRB protocol #: E-5533). Informed consent was obtained from all participants prior to their participation in the research.

## Supplementary Material

pgag186_Supplementary_Data

## Data Availability

The raw Excel data, the R Markdown cleaning and analyses code, and the qsf file for the study have been uploaded to OSF (https://osf.io/u4xav/?view_only=4f35bc0d00f44b1087c94bce46c4002c).

## Notes

a A different question was erroneously asked instead of the headline-level confidence question for the 13th headline. The value for confidence in judgment for that headline was therefore changed to be a missing value for each participant. This mistake was fixed in the Prolific replication.

## Preprints

This manuscript was posted on a preprint at https://osf.io/preprints/psyarxiv/b8w39.
