# Supplementary material for: Confident judgments of (mis)information veracity are more, rather than less, accurate
Source: PNAS Nexus. 2026 Jun 30;5(7):pgag186. doi: 10.1093/pnasnexus/pgag186 (PMC13317126; doi:10.1093/pnasnexus/pgag186)
Supplement: pgag186_Supplementary_Data [file pgag186_supplementary_data.pdf]

## Supporting Information for

# Confident judgments of (mis)information veracity are more, rather than less, accurate

Akshina Banerjee (University of Michigan - Ann Arbor), Matthew D. Rocklage (Northeastern University), Mohsen Mosleh (University of Oxford), David G. Rand (Cornell University)

Corresponding Author: Akshina Banerjee

Email: akshina@umich.edu

**OSF link:**
**https://osf.io/u4xav/overview?view_only=4f35bc0d00f44b1087c94bce46c4002c**

**This PDF file includes:**

Stimuli images

Stimuli text

Question texts

# Supplemental Materials

## Images for Confidence in General task

*Note*: The images were shown to the participants for about a second and then they were redirected to the page with the corresponding question.

(1)

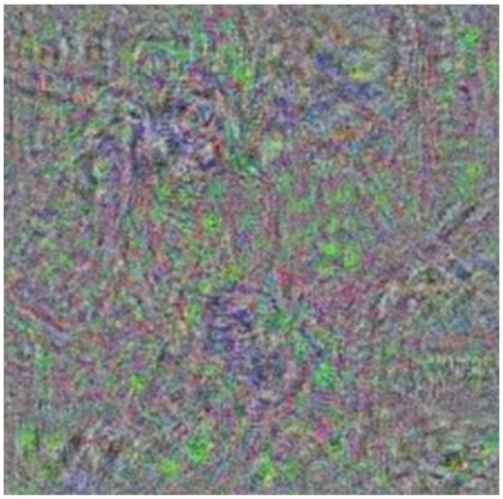

**Question**: Was that a chimpanzee or a baseball player? [Options: Chimpanzee, Baseball Player]

(2)

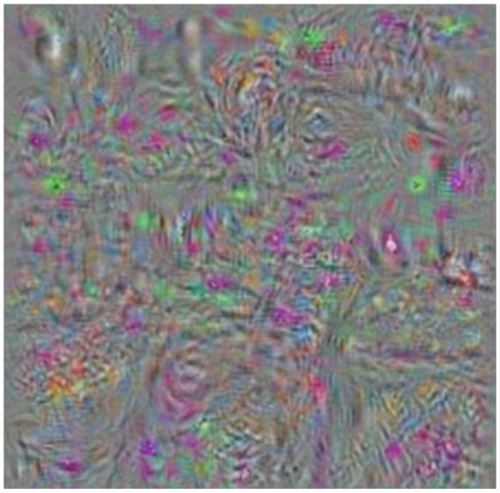

**Question**: Was that a fire station or a confectionary? [Options: Fire station, Confectionary]

(3)

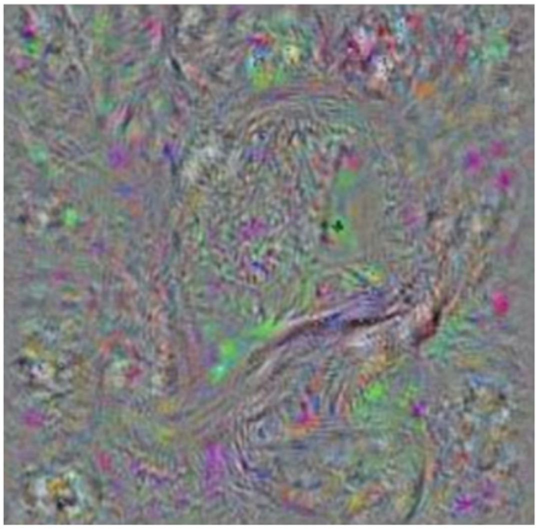

**Question**: Was that an eel or a tiger shark? [Options: Eel, Tiger shark]

(4)

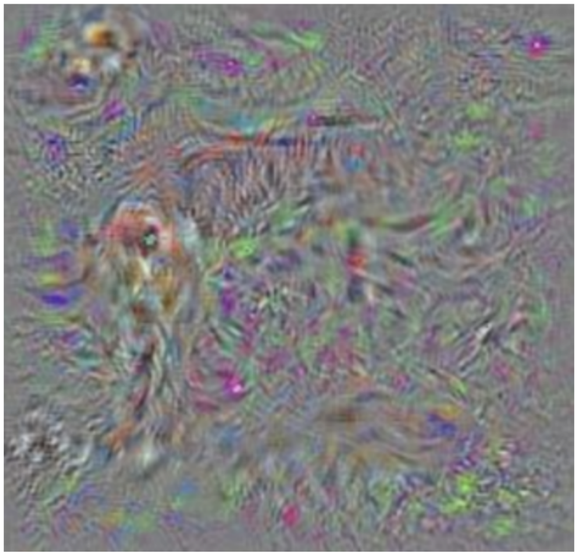

**Question**: Was that a horse or a golden retriever? [Options: Horse, Golden Retriever]

(5)

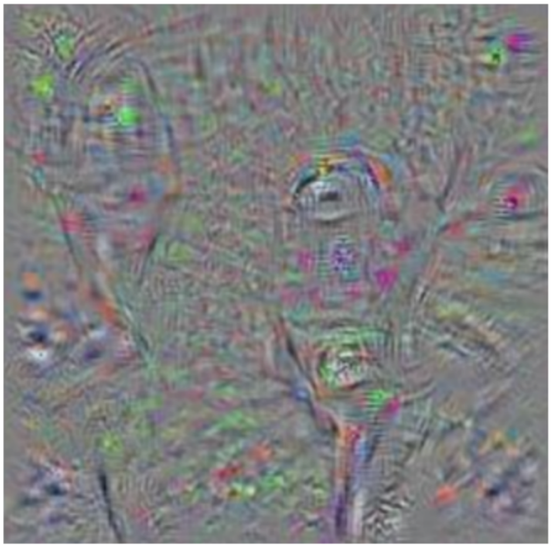

**Question**: Was that a parking meter or a stop sign? [Options: Parking meter, Stop sign]

(6)

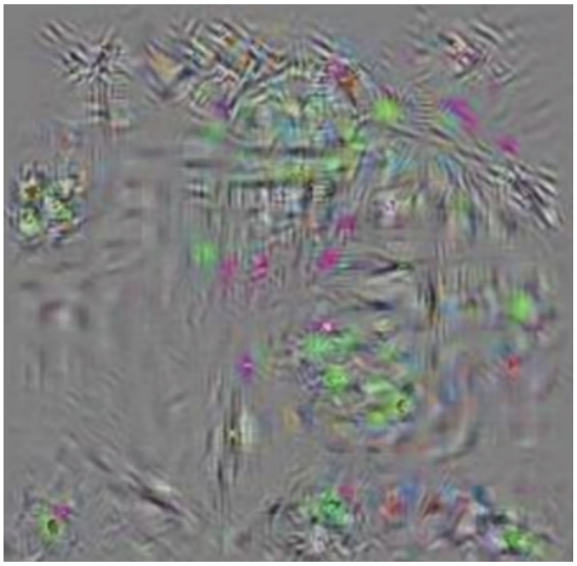

**Question**: Was that a barn or a greenhouse? [Options: Barn, Green House]

**Confidence in General Question:**

“You were just asked to identify 6 images. How many of these do you think you identified correctly?

[Please enter a number from 0 to 6 to indicate how many of the images you think you got correct.]”

**True Partisan Headlines:**

- Biden Administration cancels another $3.9 billion in student loan debt for former for-profit college students.
- Biden has tamped down talk of a primary challenge, for now.
- Biden Warns that American Values are under Assault by Trump-led Extremism
- Digital World Acquisition Corp urges Shareholders to Delay merger with Trump Media
- Ex-NYPD officer sentenced to record 10 years for Jan. 6 riot.

**False Partisan Headlines**

- Democrats Introduce Bill to 'Euthanize Seniors' to Save Social Security.
- Fauci Gets Monkey Pox at Cuomo Rave.

- 82% Pregnant Women Getting COVID Vaccine have Miscarriages - More than the Abortion Pill.
- Pentagon Study: Flu Shot Raises Risk of Coronavirus by 36% (and Other Supporting Studies).
- Pfizer Confirms COVID-Vaccinated People Can 'Shed' Spikes Proteins and Harm the Unvaccinated.

**True Non-partisan Headlines**

- More Screen Time Means Less Parent-Child Talk, Study Finds.
- Alcohol-Related Deaths Surge to Nearly 500 a Day, CDC Says.
- Lead-Tainted Applesauce Sailed Through Gaps in Food-Safety System.
- Your Inhalers and EpiPens Aren't Very Healthy for the Environment.
- Columbia Official Is Accused of Plagiarizing Dissertation From Wikipedia.

**False Non-partisan Headlines**

- Drinking Excessive Water Boosts Brain Power and Physical Performance Significantly, Study Finds.
- Eating After 8 PM Can Cause Weight Gain Regardless of Calorie Intake.
- CDC: Vitamin C Prevents Common Colds Entirely When Taken Daily.
- NASA's New Discovery Suggests that Mars Is Closer to Earth Than the Moon During Summer Months.
- World's Oldest Tree Found to Be Over 10,000 Years Younger Than Previously Thought.

**Headline Truth Judgment Question**

"Do you think this headline is true?" [Options: Yes, No]

**Confidence in Judgment Question** (displayed on the same page as the headline and the headline truth question)

"How confident are you about your answer?" [Options: Very unconfident, Somewhat unconfident, Neither unconfident nor confident, Somewhat confident, Very confident]

**Lyons et al Confidence in General Questions**

- **"How do you think you compare to other Americans in your general ability to recognize news that is made up?**

Please respond using the scale below, where 0 means you're at the very bottom (worse than 99% of people) and 100 means you're at the very top (better than 99% of people)." [Likert Scale 0-100, where 0=Worse than 99% of people, 50=Equally bad/good as others, 100=Better than 99% of people]

- "**How do you think you compare to other Americans in how well you performed in this study at recognizing news that is made up?**

  Please respond using the scale below, where 0 means you're at the very bottom (worse than 99% of people) and 100 means you're at the very top (better than 99% of people)." [Likert Scale 0-100, where 0=Worse than 99% of people, 50=Equally bad/good as others, 100=Better than 99% of people]

**Cognitive Reflection Task (Bat and Ball Problem)**

"A bat and a ball cost $1.10 in total. The bat costs $1.00 more than the ball.
How much does the ball cost?"

## Supplemental Statistical Analysis for

## Confident judgments of (mis)information veracity are more, rather than less, accurate

Lucid Sample

(1) Mixed effects model predicting headline veracity, using confidence-in-judgment (headline_confident_std), confidence-in-general (GC_std), false dummy, partisan dummy (centered), and the relevant interactions.

```
Random effects:
 Groups          Name                   Variance  Std.Dev. Corr
 person          (Intercept)            0.0233795 0.15290
                 headline_confident_std 0.0051900 0.07204  -0.26
                 false                  0.0284437 0.16865  -0.33  0.33
                 centered_partisan      0.0008751 0.02958  -0.06  0.97  0.13
 headline_number (Intercept)            0.0211018 0.14526
                 headline_confident_std 0.0008613 0.02935   0.72
                 GC_std                 0.0010731 0.03276  -0.84 -0.64
 Residual                               0.1719391 0.41466
Number of obs: 9557, groups:  person, 503; headline_number, 19

Fixed effects:
                                              Estimate Std. Error        df t value Pr(>|t|)
(Intercept)                                   0.630236   0.049354 15.602202  12.770 1.14e-09 ***
headline_confident_std                        0.081758   0.012760 20.129120   6.407 2.90e-06 ***
GC_std                                       -0.010939   0.014391 25.474706  -0.760  0.45417
false                                        -0.255096   0.067748 15.363737  -3.765  0.00180 **
centered_partisan                             0.061315   0.155345 14.976345   0.395  0.69863
headline_confident_std:false                 -0.096108   0.016650 16.231641  -5.772 2.70e-05 ***
GC_std:false                                  0.066396   0.018894 21.080793   3.514  0.00205 **
headline_confident_std:centered_partisan      0.046871   0.037060 14.045911   1.265  0.22655
GC_std:centered_partisan                      0.001152   0.040006 15.022668   0.029  0.97740
false:centered_partisan                      -0.164340   0.219007 14.975052  -0.750  0.46465
GC_std:false:centered_partisan               -0.005767   0.056372 14.991024  -0.102  0.91987
headline_confident_std:false:centered_partisan -0.060475   0.051847 13.620437  -1.166  0.26346
---
Signif. codes:  0 ‘***’ 0.001 ‘**’ 0.01 ‘*’ 0.05 ‘.’ 0.1 ‘ ’ 1
```

(2) Mixed effects model predicting headline veracity, using confidence-in-judgment (headline_confident_std), confidence-in-general (GC_std), partisan dummy (centered), and the relevant interactions for true headlines.

```
Random effects:
 Groups          Name                    Variance  Std.Dev. Corr
 person          (Intercept)             0.0227034 0.15068
                 headline_confident_std  0.0063447 0.07965  -0.28
                 centered_partisan       0.0013001 0.03606  -0.63 -0.56
 headline_number (Intercept)             0.0166862 0.12917
                 headline_confident_std  0.0003314 0.01820   0.75
                 GC_std                  0.0007278 0.02698  -0.80 -0.20
 Residual                                0.1795750 0.42376
Number of obs: 4527, groups:  person, 503; headline_number, 9

Fixed effects:
                                          Estimate Std. Error        df t value Pr(>|t|)
(Intercept)                              0.6323895  0.0441048  7.3448352  14.338 1.23e-06 ***
headline_confident_std                   0.0903296  0.0105385 15.8197520   8.571 2.44e-07 ***
GC_std                                  -0.0128707  0.0130526 14.3843051  -0.986    0.340
centered_partisan                        0.0612141  0.1385049  6.9857952   0.442    0.672
headline_confident_std:centered_partisan  0.0463634  0.0282846  8.2145874   1.639    0.139
GC_std:centered_partisan                 0.0006768  0.0350859  7.4061521   0.019    0.985
---
Signif. codes:  0 ‘***’ 0.001 ‘**’ 0.01 ‘*’ 0.05 ‘.’ 0.1 ‘ ’ 1
```

(3) Mixed effects model predicting headline veracity, using confidence-in-judgment (headline_confident_std), confidence-in-general (GC_std), partisan dummy (centered), and the relevant interactions for false headlines.

```
Random effects:
 Groups          Name                   Variance Std.Dev. Corr
 person          (Intercept)            0.035168 0.18753
                 headline_confident_std 0.007231 0.08503  0.19
                 centered_partisan      0.003792 0.06158  0.55  0.93
 headline_number (Intercept)            0.024507 0.15655
                 headline_confident_std 0.001288 0.03589   0.64
                 GC_std                 0.001390 0.03728  -0.83 -0.80
 Residual                               0.162322 0.40289
Number of obs: 5030, groups:  person, 503; headline_number, 10

Fixed effects:
                                         Estimate Std. Error       df t value Pr(>|t|)
(Intercept)                               0.37563    0.05058  8.47753   7.427 5.48e-05 ***
headline_confident_std                   -0.01884    0.01394 10.85416  -1.352  0.20392
GC_std                                    0.05426    0.01571 16.34630   3.453  0.00319 **
centered_partisan                        -0.10329    0.16615  7.99785  -0.622  0.55148
headline_confident_std:centered_partisan -0.01620    0.04216  7.37423  -0.384  0.71158
GC_std:centered_partisan                 -0.00746    0.04378  8.02526  -0.170  0.86889
---
Signif. codes:  0 ‘***’ 0.001 ‘**’ 0.01 ‘*’ 0.05 ‘.’ 0.1 ‘ ’ 1
```

(4) Mixed effects model predicting headline veracity, using confidence-in-judgment (headline_confident_std), Lyons et al. confidence-in-general (OGC_std), false dummy, partisan dummy (centered), and the relevant interactions.

```
Random effects:
 Groups          Name                  Variance  Std.Dev. Corr
 person          (Intercept)           0.0233527 0.15282
                 headline_confident_std 0.0052500 0.07246  -0.26
                 false                 0.0317585 0.17821  -0.35  0.32
                 centered_partisan     0.0008231 0.02869  -0.01  0.97  0.22
 headline_number (Intercept)           0.0209925 0.14489
                 headline_confident_std 0.0010345 0.03216   0.71
                 OGC_std               0.0003608 0.01899  -0.39 -0.73
 Residual                              0.1725299 0.41537
Number of obs: 9557, groups:  person, 503; headline_number, 19

Fixed effects:
                                              Estimate Std. Error        df t value Pr(>|t|)
(Intercept)                                   0.629991   0.049233 15.608040  12.796 1.10e-09 ***
headline_confident_std                        0.082371   0.013567 19.523891   6.072 6.87e-06 ***
OGC_std                                       0.007618   0.011389 37.331811   0.669  0.50772
false                                        -0.253767   0.067629 15.412022  -3.752  0.00184 **
centered_partisan                             0.061571   0.154953 14.977438   0.397  0.69671
headline_confident_std:false                 -0.100980   0.017822 16.167758  -5.666 3.37e-05 ***
OGC_std:false                                 0.029157   0.014737 29.429441   1.978  0.05731 .
headline_confident_std:centered_partisan      0.050131   0.040014 14.482871   1.253  0.23012
OGC_std:centered_partisan                    -0.016888   0.028839 15.569115  -0.586  0.56654
false:centered_partisan                      -0.165538   0.218455 14.976258  -0.758  0.46035
OGC_std:false:centered_partisan              -0.016800   0.040494 15.318262  -0.415  0.68399
headline_confident_std:false:centered_partisan -0.055511   0.055942 14.004971  -0.992  0.33788
---
Signif. codes:  0 ‘***’ 0.001 ‘**’ 0.01 ‘*’ 0.05 ‘.’ 0.1 ‘ ’ 1
```

(5) Mixed effects model predicting headline veracity, using confidence-in-judgment (headline_confident_std), Lyons et al. confidence-in-general (OGC_std), partisan dummy (centered), and the relevant interactions for true headlines.

```
Random effects:
 Groups          Name                   Variance  Std.Dev. Corr
 person          (Intercept)            2.259e-02 0.150310
                 headline_confident_std 6.474e-03 0.080461 -0.26
                 centered_partisan      1.252e-03 0.035383 -0.63 -0.58
 headline_number (Intercept)            1.654e-02 0.128623
                 headline_confident_std 3.151e-04 0.017752  0.89
                 OGC_std                9.886e-05 0.009943 -0.82 -0.99
 Residual                               1.802e-01 0.424479
Number of obs: 4527, groups:  person, 503; headline_number, 9

Fixed effects:
                                          Estimate Std. Error        df t value Pr(>|t|)
(Intercept)                               0.631802   0.043925  7.355332  14.384 1.19e-06 ***
headline_confident_std                    0.089740   0.010552 13.066097   8.504 1.10e-06 ***
OGC_std                                   0.007788   0.010128 42.234688   0.769    0.446
centered_partisan                         0.061966   0.137931  6.993960   0.449    0.667
headline_confident_std:centered_partisan  0.049601   0.028560  7.021688   1.737    0.126
OGC_std:centered_partisan                -0.016431   0.023623 13.065389  -0.696    0.499
---
Signif. codes:  0 ‘***’ 0.001 ‘**’ 0.01 ‘*’ 0.05 ‘.’ 0.1 ‘ ’ 1
```

(6) Mixed effects model predicting headline veracity, using confidence-in-judgment (headline_confident_std), Lyons et al. confidence-in-general (OGC_std), partisan dummy (centered), and the relevant interactions for false headlines.

```
Random effects:
 Groups          Name                   Variance  Std.Dev. Corr
 person          (Intercept)            0.0367713 0.19176
                 headline_confident_std 0.0073899 0.08596  0.24
                 centered_partisan      0.0042333 0.06506  0.57  0.93
 headline_number (Intercept)            0.0242583 0.15575
                 headline_confident_std 0.0014512 0.03809   0.62
                 OGC_std                0.0006294 0.02509  -0.23 -0.65
 Residual                               0.1627908 0.40347
Number of obs: 5030, groups:  person, 503; headline_number, 10

Fixed effects:
                                         Estimate Std. Error       df t value Pr(>|t|)
(Intercept)                               0.37664    0.05037  8.50365   7.478 5.13e-05 ***
headline_confident_std                   -0.02314    0.01456 10.58211  -1.590   0.1412
OGC_std                                   0.03502    0.01324 25.27701   2.645   0.0138 *
centered_partisan                        -0.10410    0.16532  7.99750  -0.630   0.5465
headline_confident_std:centered_partisan -0.00652    0.04447  7.48591  -0.147   0.8873
OGC_std:centered_partisan                -0.03712    0.03306  8.11000  -1.123   0.2935
---
Signif. codes:  0 ‘***’ 0.001 ‘**’ 0.01 ‘*’ 0.05 ‘.’ 0.1 ‘ ’ 1
```

(7) Mixed effects model predicting headline veracity, using confidence-in-judgment (headline_confident_std), confidence-in-general (GC_std), false dummy, partisan dummy (centered), and the relevant interactions for Democrat-leaning participants.

```
Random effects:
 Groups          Name                   Variance  Std.Dev. Corr
 person          (Intercept)            0.0216983 0.14730
                 headline_confident_std 0.0072284 0.08502  -0.18
                 false                  0.0286880 0.16938  -0.31  0.36
                 centered_partisan      0.0002377 0.01542   0.30  0.83 -0.11
 headline_number (Intercept)            0.0240752 0.15516
                 headline_confident_std 0.0012916 0.03594   0.59
                 GC_std                 0.0003746 0.01935  -0.71 -0.68
 Residual                               0.1645078 0.40560
Number of obs: 5738, groups:  person, 302; headline_number, 19

Fixed effects:
                                               Estimate Std. Error       df t value Pr(>|t|)
(Intercept)                                     0.64014    0.05309 15.84111  12.058 2.16e-09 ***
headline_confident_std                          0.09201    0.01616 21.77199   5.693 1.04e-05 ***
GC_std                                         -0.01180    0.01359 42.05297  -0.868 0.390295
false                                          -0.27578    0.07282 15.55073  -3.787 0.001690 **
centered_partisan                               0.05567    0.16647 14.97744   0.334 0.742720
headline_confident_std:false                   -0.10517    0.02065 16.27285  -5.092 0.000103 ***
GC_std:false                                    0.06721    0.01721 30.90815   3.906 0.000476 ***
headline_confident_std:centered_partisan        0.04718    0.04617 14.33372   1.022 0.323841
GC_std:centered_partisan                        0.02892    0.03253 14.73230   0.889 0.388284
false:centered_partisan                        -0.18713    0.23469 14.97628  -0.797 0.437711
GC_std:false:centered_partisan                 -0.04202    0.04585 14.72068  -0.916 0.374272
headline_confident_std:false:centered_partisan -0.06878    0.06424 13.59920  -1.071 0.302977
---
Signif. codes:  0 ‘***’ 0.001 ‘**’ 0.01 ‘*’ 0.05 ‘.’ 0.1 ‘ ’ 1
```

(8) Mixed effects model predicting headline veracity, using confidence-in-judgment (headline_confident_std), confidence-in-general (GC_std), false dummy, partisan dummy (centered), and the relevant interactions for Republican-leaning participants.

```
Random effects:
 Groups          Name                   Variance  Std.Dev. Corr
 person          (Intercept)            0.0261882 0.16183
                 headline_confident_std 0.0019054 0.04365  -0.58
                 false                  0.0283281 0.16831  -0.35  0.40
                 centered_partisan      0.0070242 0.08381  -0.11  0.85  0.07
 headline_number (Intercept)            0.0169398 0.13015
                 headline_confident_std 0.0005269 0.02296   1.00
                 GC_std                 0.0031183 0.05584  -0.83 -0.83
 Residual                               0.1806114 0.42498
Number of obs: 3819, groups:  person, 201; headline_number, 19

Fixed effects:
                                           Estimate Std. Error       df t value Pr(>|t|)
(Intercept)                                 0.61666    0.04602 16.96428  13.401 1.88e-10 ***
headline_confident_std                      0.06714    0.01450 33.38174   4.631 5.33e-05 ***
GC_std                                     -0.01262    0.02390 24.15848  -0.528  0.60225
false                                      -0.22385    0.06255 16.06370  -3.579  0.00249 **
centered_partisan                           0.07095    0.14183 14.98363   0.500  0.62418
headline_confident_std:false               -0.08796    0.01891 27.63907  -4.652 7.38e-05 ***
GC_std:false                                0.06706    0.03136 20.03319   2.139  0.04495 *
headline_confident_std:centered_partisan    0.04599    0.04012 19.63402   1.146  0.26542
GC_std:centered_partisan                   -0.03749    0.06739 15.23486  -0.556  0.58614
false:centered_partisan                    -0.13115    0.19978 14.93267  -0.656  0.52153
GC_std:false:centered_partisan              0.04819    0.09464 15.01045   0.509  0.61804
headline_confident_std:false:centered_partisan -0.04278    0.05630 19.31509  -0.760  0.45653
---
Signif. codes:  0 ‘***’ 0.001 ‘**’ 0.01 ‘*’ 0.05 ‘.’ 0.1 ‘ ’ 1
```

(9) Mixed effects model predicting headline veracity, using confidence-in-judgment (headline_confident_std), confidence-in-general (GC_std), false dummy, partisan dummy (centered), dummy for getting CRT correct (crt_correct), and the relevant interactions.

```
Random effects:
 Groups          Name                  Variance  Std.Dev. Corr
 person          (Intercept)           0.0235319 0.15340
                 headline_confident_std 0.0051229 0.07157  -0.25
                 false                 0.0282372 0.16804  -0.34  0.33
                 centered_partisan     0.0009086 0.03014  -0.11  0.98  0.14
 headline_number (Intercept)           0.0210519 0.14509
                 headline_confident_std 0.0008701 0.02950   0.72
                 GC_std                0.0010576 0.03252  -0.84 -0.64
 Residual                              0.1718969 0.41460
Number of obs: 9557, groups:  person, 503; headline_number, 19

Fixed effects:
                                              Estimate Std. Error        df t value Pr(>|t|)
(Intercept)                                  6.447e-01  5.097e-02  1.784e+01  12.650 2.41e-10 ***
headline_confident_std                       6.100e-02  1.697e-02  6.064e+01   3.595 0.000653 ***
GC_std                                      -2.134e-02  1.887e-02  7.269e+01  -1.131 0.261882
false                                       -2.352e-01  6.949e-02  1.710e+01  -3.385 0.003495 **
centered_partisan                            6.154e-02  1.552e-01  1.500e+01   0.397 0.697224
crt_correct                                 -2.056e-02  1.971e-02  4.899e+02  -1.043 0.297303
headline_confident_std:false                -7.566e-02  2.130e-02  4.283e+01  -3.552 0.000944 ***
GC_std:false                                 8.080e-02  2.403e-02  5.452e+01   3.362 0.001420 **
headline_confident_std:centered_partisan     4.622e-02  3.719e-02  1.406e+01   1.243 0.234310
GC_std:centered_partisan                     1.299e-03  3.979e-02  1.505e+01   0.033 0.974391
false:centered_partisan                     -1.648e-01  2.188e-01  1.499e+01  -0.754 0.462782
false:crt_correct                           -3.204e-02  2.405e-02  4.946e+02  -1.332 0.183375
headline_confident_std:crt_correct           3.150e-02  1.685e-02  9.300e+02   1.870 0.061861 .
GC_std:crt_correct                           1.673e-02  1.945e-02  4.968e+02   0.860 0.390089
GC_std:false:centered_partisan              -5.813e-03  5.606e-02  1.501e+01  -0.104 0.918786
headline_confident_std:false:centered_partisan -5.983e-02  5.203e-02  1.363e+01  -1.150 0.269993
headline_confident_std:false:crt_correct    -3.211e-02  2.009e-02  5.375e+03  -1.598 0.110095
GC_std:false:crt_correct                    -2.487e-02  2.365e-02  4.953e+02  -1.052 0.293377
---
Signif. codes:  0 ‘***’ 0.001 ‘**’ 0.01 ‘*’ 0.05 ‘.’ 0.1 ‘ ’ 1
```

(10) Mixed effects model predicting headline veracity, using confidence-in-judgment (headline_confident_std), person level average of confidence-in-judgment (hc_person_mean), confidence-in-general (GC_std), false dummy, partisan dummy (centered), and the relevant interactions.

```
Random effects:
 Groups          Name                  Variance  Std.Dev. Corr
 person          (Intercept)           0.0235869 0.15358
                 headline_confident_std 0.0050573 0.07111  -0.25
                 false                 0.0282812 0.16817  -0.34  0.32
                 centered_partisan     0.0008289 0.02879  -0.01  0.96  0.15
 headline_number (Intercept)           0.0211405 0.14540
                 headline_confident_std 0.0008563 0.02926   0.72
                 GC_std                0.0010722 0.03274  -0.84 -0.64
 Residual                              0.1719984 0.41473
Number of obs: 9557, groups:  person, 503; headline_number, 19

Fixed effects:
                                              Estimate Std. Error         df t value Pr(>|t|)
(Intercept)                                   0.629759   0.049402  15.619406  12.748 1.15e-09 ***
headline_confident_std                        0.077137   0.013000  21.896959   5.934 5.80e-06 ***
hc_person_mean                                0.027138   0.015593 721.995961   1.740  0.08221 .
GC_std                                       -0.012139   0.014419  25.729912  -0.842  0.40762
false                                        -0.254658   0.067806  15.373594  -3.756  0.00184 **
centered_partisan                             0.061673   0.155485  14.988215   0.397  0.69722
headline_confident_std:false                 -0.094966   0.016631  16.315952  -5.710 2.99e-05 ***
GC_std:false                                  0.066506   0.018882  21.074094   3.522  0.00202 **
headline_confident_std:centered_partisan      0.046907   0.036986  14.072859   1.268  0.22529
GC_std:centered_partisan                      0.001372   0.039993  15.037131   0.034  0.97308
false:centered_partisan                      -0.165145   0.219206  14.987119  -0.753  0.46289
GC_std:false:centered_partisan               -0.005861   0.056355  15.006223  -0.104  0.91855
headline_confident_std:false:centered_partisan -0.060337   0.051743  13.645090  -1.166  0.26356
---
Signif. codes:  0 ‘***’ 0.001 ‘**’ 0.01 ‘*’ 0.05 ‘.’ 0.1 ‘ ’ 1
```

Prolific Sample

Mixed effects model predicting headline veracity, using confidence-in-judgment (headline_confident_std), confidence-in-general (GC_std), false dummy, partisan dummy (centered), and the relevant interactions.

```
Random effects:
 Groups          Name                  Variance  Std.Dev. Corr
 person          (Intercept)           0.0247891 0.15745
                 headline_confident_std 0.0046898 0.06848  -0.35
                 false                 0.0325827 0.18051  -0.73  0.17
                 centered_partisan     0.0077360 0.08795  -0.20  0.36  0.22
 headline_number (Intercept)           0.0259187 0.16099
                 headline_confident_std 0.0007236 0.02690   0.78
                 GC_std                0.0005699 0.02387  -0.81 -0.83
 Residual                              0.1544245 0.39297
Number of obs: 9960, groups:  person, 498; headline_number, 20

Fixed effects:
                                              Estimate Std. Error         df t value Pr(>|t|)
(Intercept)                                  0.6659664  0.0517386 16.6034207  12.872 4.63e-10 ***
headline_confident_std                       0.0815278  0.0115004 22.3924322   7.089 3.73e-07 ***
GC_std                                       0.0109796  0.0117914 37.9620035   0.931    0.358
false                                       -0.3766973  0.0729305 16.3871383  -5.165 8.71e-05 ***
centered_partisan                            0.0003904  0.1707866 15.9682461   0.002    0.998
headline_confident_std:false                -0.1334319  0.0153330 17.7200416  -8.702 8.21e-08 ***
GC_std:false                                 0.0162203  0.0156674 29.9437456   1.035    0.309
headline_confident_std:centered_partisan     0.0322991  0.0353138 16.2050937   0.915    0.374
GC_std:centered_partisan                    -0.0184517  0.0316685 16.4315292  -0.583    0.568
false:centered_partisan                     -0.1000051  0.2414728 15.9534882  -0.414    0.684
GC_std:false:centered_partisan               0.0123600  0.0444205 15.9219352   0.278    0.784
headline_confident_std:false:centered_partisan -0.0284161  0.0495341 15.6849073  -0.574    0.574
---
Signif. codes:  0 ‘***’ 0.001 ‘**’ 0.01 ‘*’ 0.05 ‘.’ 0.1 ‘ ’ 1
```
